# Job satisfaction and its correlation with pharmacists’ performance and patient trust

**DOI:** 10.3389/fmed.2025.1624990

**Published:** 2025-07-25

**Authors:** Sultan M. Alshahrani, Azfar A. Ishaqui, Sirajudeen Shiak Alavudeen

**Affiliations:** Department of Clinical Pharmacy, College of Pharmacy, King Khalid University, Abha, Saudi Arabia

**Keywords:** systematic review, meta-analysis, job satisfaction, pharmacists’ performance, patient trust, healthcare workers’ satisfaction, pharmacist job satisfaction, pharmacy practice

## Abstract

**Background:**

Job satisfaction is a key factor influencing pharmacists’ performance and the level of trust patients place in them. Recognizing the relationship between these elements is essential for enhancing pharmacy services, improving patient outcomes, and creating a supportive work environment in healthcare settings.

**Objective:**

The purpose of this study is to assess how pharmacists’ job satisfaction relates to their professional performance and the level of trust patients place in them. This review aims to provide evidence-based insights into how these factors collectively influence healthcare outcomes.

**Methods:**

We conducted a comprehensive systematic literature search across several electronic databases—PubMed, Scopus, Web of Science, Embase, and Cochrane Library—covering publications up to December 2024. Our search strategy effectively identified relevant studies exploring job satisfaction among pharmacists and its impact on work performance and patient trust. We utilized Medical Subject Headings (MeSH) terms along with targeted free-text keywords such as “pharmacist job satisfaction,” “work performance,” “patient trust,” “pharmacy practice,” and “professional commitment.” By employing Boolean operators (AND, OR), we refined and enhanced our results. To assess the risk of bias, we used the Cochrane Risk of Bias Tool for randomized controlled trials and the ROBINS-I tool for non-randomized studies. This review was registered on PROSPERO under registration number CRD42024627893.

**Results:**

A total of 65 studies were included in the final analysis, representing a variety of geographic regions and pharmacist populations. The meta-analysis revealed that job satisfaction among pharmacists was significantly linked to improved professional performance and increased patient trust. Monthly income and workplace environment emerged as the most prominent predictors, followed by years of experience and professional role. A fixed-effects model was utilized due to low to moderate heterogeneity (I^2^ < 50%), and the results remained robust under sensitivity analysis. Additionally, subgroup analysis of pharmacist-related factors further confirmed the reliability of these associations. Funnel plot symmetry and statistical tests indicated minimal publication bias.

**Conclusion:**

This review highlights a significant link between job satisfaction, pharmacists’ performance, and patient trust. Higher job satisfaction correlates with better pharmacist engagement and patient outcomes. However, many pharmacists intend to leave due to job dissatisfaction. Targeted improvements in workplace culture, compensation, and recognition could significantly enhance pharmacist retention and patient-centered care.

**Systematic review registration:**

Identifier [CRD2024627893] RD42024627893. https://www.crd.york.ac.uk/PROSPERO/view/CRD42024627893.

## Highlights

- This study synthesizes data from a diverse array of high-quality sources, facilitating a comprehensive examination of the interrelations among job satisfaction, pharmacist performance, and patient trust.
- We employed a rigorous methodology encompassing a systematic search strategy, eligibility criteria for screening studies, quality assessment of the selected literature, and a meta-analytic approach, which enhances the reliability of our findings.
- The inclusion of studies from various healthcare contexts and geographic locations increases the generalizability of our results across different settings.
- Furthermore, we implemented strategies to detect publication bias and conducted sensitivity analyses, bolstering the robustness of our conclusions.
- Nevertheless, several limitations warrant attention. Variability in study designs, the operationalization of job satisfaction, and the heterogeneity of pharmacy environments may introduce inconsistencies in the aggregated results.
- A significant proportion of the studies included are cross-sectional, limiting our ability to infer causality between job satisfaction and changes in pharmacist performance. Additionally, many studies relied on self-reported measures, which raises concerns about response bias. There is also the possibility of residual publication bias, suggesting that non-significant findings may be underreported in the existing literature. Future research should prioritize longitudinal studies and standardized job satisfaction measures to deepen understanding and strengthen evidence for policy development.

## Introduction

Pharmacists play a vital role in global healthcare systems, acting as essential gatekeepers in the safe, effective, and patient-centered management of pharmacotherapy. Their responsibilities extend beyond traditional dispensing duties to include patient counseling, medication therapy management, and involvement in public health initiatives (1, 2). As frontline healthcare providers, pharmacists significantly influence patient outcomes, medication adherence, and the overall quality of healthcare delivery (3).

Job satisfaction within the pharmacy profession is a crucial factor that affects pharmacists’ professional performance, motivation, and the quality of their interactions with patients (4). Research consistently shows that higher job satisfaction correlates with improved performance metrics, while dissatisfaction can lead to negative outcomes that compromise both pharmacist wellbeing and the quality of care delivered (5).

Several factors contribute to job satisfaction in pharmacy practice. These include workload management—particularly in light of increasing prescription demands—work environment quality, professional autonomy, equitable compensation, opportunities for career advancement, and interprofessional relationships (6). Pharmacists who work in supportive and equitable environments tend to exhibit greater professional engagement, better decision-making abilities, and stronger communication skills (7). This sense of professional fulfillment enhances patient trust, medication adherence, and overall health outcomes (8). In contrast, job dissatisfaction is often linked to burnout, staff turnover, and diminished care quality (9).

Trust is a fundamental element of effective healthcare delivery, especially in pharmacy settings, where pharmacists are among the most accessible healthcare providers (9). Trust is influenced by various factors, including a pharmacist’s communication style, clinical competence, perceived empathy, and level of job satisfaction (10, 11). Pharmacists who experience high job satisfaction are more likely to engage in genuine, patient-centered interactions, which fosters trust and improves health outcomes (12).

Despite growing awareness of the importance of job satisfaction in healthcare, there is still a lack of comprehensive evaluation concerning its impact on pharmacist performance and the trust patients have in them (11, 13, 14). While various individual studies have explored these relationships, no prior synthesis has thoroughly addressed the interconnectedness of job satisfaction, performance, and trust within a unified framework (15).

This review aims to answer the following question: What is the relationship between pharmacists’ job satisfaction, their professional performance, and the trust that patients place in them?

To address this, we conducted a systematic review and meta-analysis to synthesize current literature examining how job satisfaction influences pharmacists’ ability to deliver high-quality (16–18), patient-centered care and build strong therapeutic relationships (19–21). These insights can support policymakers and healthcare leaders in designing interventions to enhance both pharmacy practice and patient trust (22–24).

**Figure 1:**
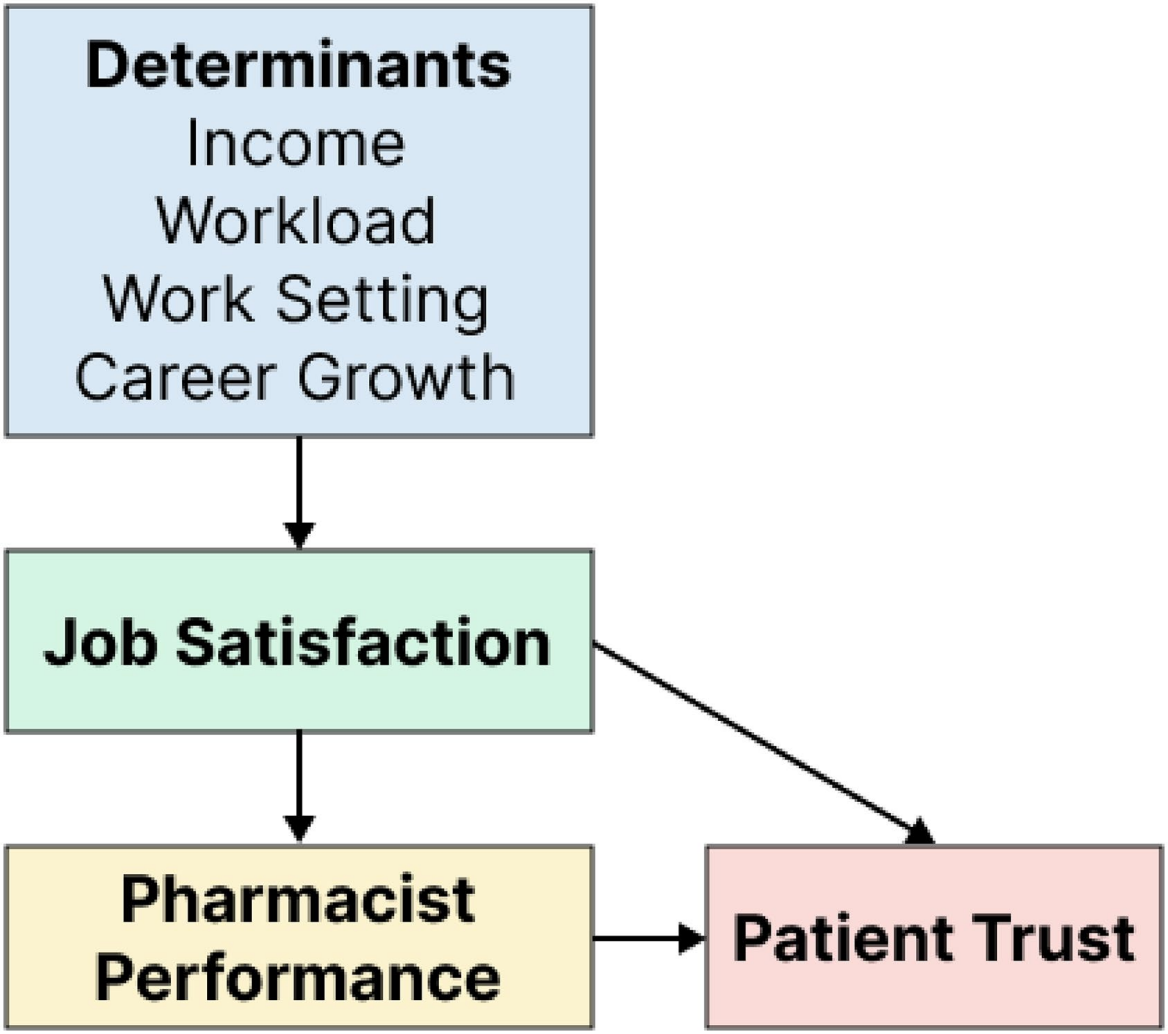
Conceptual model linking pharmacist job satisfaction to performance and patient trust.

A conceptual model illustrating the theoretical linkages between job satisfaction, pharmacists’ performance, and patient trust is presented in Figure 1. The model proposes that pharmacist-specific and organizational factors influence job satisfaction, which, in turn, affects performance outcomes and the level of trust patients experience.

## Methods

### Study design and research question

This study is a systematic review and meta-analysis aimed at examining the relationship between pharmacists’ job satisfaction, their professional performance, and the trust that patients have in them. The guiding research question for this review is:

What is the relationship between pharmacists’ job satisfaction, their professional performance, and the trust that patients place in them?

### Protocol and registration

This review follows the guidelines established by the Preferred Reporting Items for Systematic Reviews and Meta-Analyses (PRISMA). It is based on a registered protocol with the International Prospective Register of Systematic Reviews (PROSPERO) (Ref: CRD42024627893). The protocol outlines the objectives, eligibility criteria, data sources, methods for quality assessment, and strategies for data synthesis in detail.

### Data sources and searches

We kicked off a thorough literature search using a variety of electronic databases, including PubMed, Scopus, Web of Science, Embase, Cochrane Library, and Google Scholar for any gray literature and additional findings. Our search looked at publications up until December 2024 and included a mix of Medical Subject Headings (MeSH) and some key terms such as “pharmacist job satisfaction,” “work performance,” “patient trust,” “pharmacy practice,” and “professional commitment”.

To make our search a bit more precise, we employed Boolean operators (AND, OR). If you are interested in the details, the complete search strategy for each database can be found in the Supplementary Material. Additionally, we had two independent reviewers go through all the titles, abstracts, and full texts. If there were any disagreements, we simply held a discussion or brought in a third reviewer to help out.

### Eligibility criteria

The inclusion criteria were designed to capture studies that rigorously explored the nexus between job satisfaction among pharmacists, their work performance, and the trust patients place in them. Eligible studies employed quantitative, qualitative, or mixed methodologies and provided quantifiable data pertinent to these constructs. Only peer-reviewed articles published in English were considered to ensure methodological integrity and accessibility. Moreover, studies are needed to present definitive findings on how pharmacist job satisfaction affects professional performance and patient trust across various pharmacy environments.

In contrast, studies were excluded if they did not focus explicitly on job satisfaction within the pharmacist demographic or primarily addressed other healthcare professionals without establishing clear links to pharmacy practice. Research that lacked empirical evidence—including opinion pieces, commentaries, or editorials—was also disregarded. Additionally, to uphold methodological rigor and relevance, we excluded duplicate studies, conference abstracts, unpublished dissertations, and articles that did not yield measurable outcomes related to work performance or patient trust.

Only studies with extractable numerical data were deemed eligible for the meta-analysis, despite the fact that the review contained both quantitative and qualitative studies. To offer contextual insights, only qualitative studies or the qualitative elements of mixed-methods studies were incorporated into the narrative synthesis; however, they were not included in the statistical pooling of results.

Although one of the included studies was itself a systematic review, it was kept in our analysis because of its distinct thematic synthesis, which none of the primary studies we included were able to replicate. We compared its listed primary sources with our dataset to prevent duplication and eliminated any overlapping data from additional analysis. When systematic reviews provide synthesized insights pertinent to the current review’s goals, they may be included in accordance with PRISMA guidelines, as long as duplication is openly managed.

### Data extraction and management

Data extraction was conducted independently by two reviewers utilizing a standardized, pilot-tested extraction form tailored for this systematic review. The extraction process encompassed essential study attributes, including author names, publication year, country of origin, study design, and sample size, as well as demographic data of the participants. Furthermore, we meticulously gathered detailed information regarding the measurement of job satisfaction, indicators of pharmacist performance, and constructs related to patient trust. Effect sizes, statistical results, and relevant quantitative and qualitative findings were also recorded. Any discrepancies between the reviewers were resolved through thorough discussion, and, if needed, a third reviewer was involved to achieve consensus. This methodical approach reinforced the consistency, transparency, and accuracy of our data collection procedures.

### Quality assessment

To evaluate the methodological rigor of the included studies, we used validated tools that align with the methodological standards set by the Frontiers journal series. For observational studies, we applied the Newcastle-Ottawa Scale (NOS) to assess participant selection, study comparability, and outcome measurement. For randomized controlled trials (RCTs), we utilized the Cochrane Risk of Bias 2 (RoB 2) tool, which evaluates factors such as randomization procedures, allocation concealment, blinding, and completeness of outcome data. Each study was classified as low, moderate, or high risk of bias to ensure confidence in the synthesized evidence regarding the relationship between pharmacists’ job satisfaction, work performance, and patient trust.

### Data synthesis and statistical analysis

We synthesized data from the included studies using quantitative meta-analytic methods. Statistical analyses were performed using Review Manager (RevMan) software version 5.4. For dichotomous outcomes, we calculated odds ratios (ORs) along with their corresponding 95% confidence intervals (CIs). In instances where continuous data were available, we computed mean differences (MDs) or standardized mean differences (SMDs) with 95% CIs as applicable.

### Assessment of heterogeneity, publication bias, and sensitivity analysis

To evaluate heterogeneity among the studies, we employed the chi-squared test (χ^2^) and quantified it using the I^2^ statistic. We defined the I^2^ thresholds as follows: 25% for low heterogeneity, 50% for moderate heterogeneity, and 75% for high heterogeneity. We opted for a fixed-effects model for both the meta-analysis and sensitivity analysis since the studies demonstrated low to moderate heterogeneity across the primary outcome measures (I^2^ < 50%). Moreover, the studies were largely comparable in terms of population characteristics, types of interventions, and outcome definitions. This methodological consistency and the observed statistical homogeneity justified our use of a fixed-effects model to produce precise and reliable pooled estimates, thereby negating the need for additional random-effects modeling.

We assessed publication bias using funnel plots and, when suitable, applied Egger’s regression test. Additionally, we conducted a sensitivity analysis to evaluate the robustness of the pooled estimates by excluding outliers or studies deemed to be at high risk of bias.

All analyses adhered to PRISMA guidelines and followed established best practices for conducting systematic reviews and meta-analyses.

## Results

### Identification and selection of studies

We conducted a comprehensive literature review to explore the relationships among job satisfaction, pharmacists’ performance, and patient trust. Our search yielded an initial pool of 3,820 articles across various databases, supplemented by 95 additional studies from alternative sources. After systematically removing duplicates, we proceeded with 2,940 unique studies. In the title and abstract screening phase, we excluded 2,610 articles that did not meet our predefined criteria. Subsequently, we undertook a thorough evaluation of the full texts of 330 studies. After careful consideration, 145 studies were excluded for several reasons: 85 were not aligned with our outcomes of interest, 42 fell outside the scope of our investigation, 10 presented insufficient data for analysis, 5 demonstrated methodological flaws, and 3 were identified as duplicate publications.

**Figure 2:**
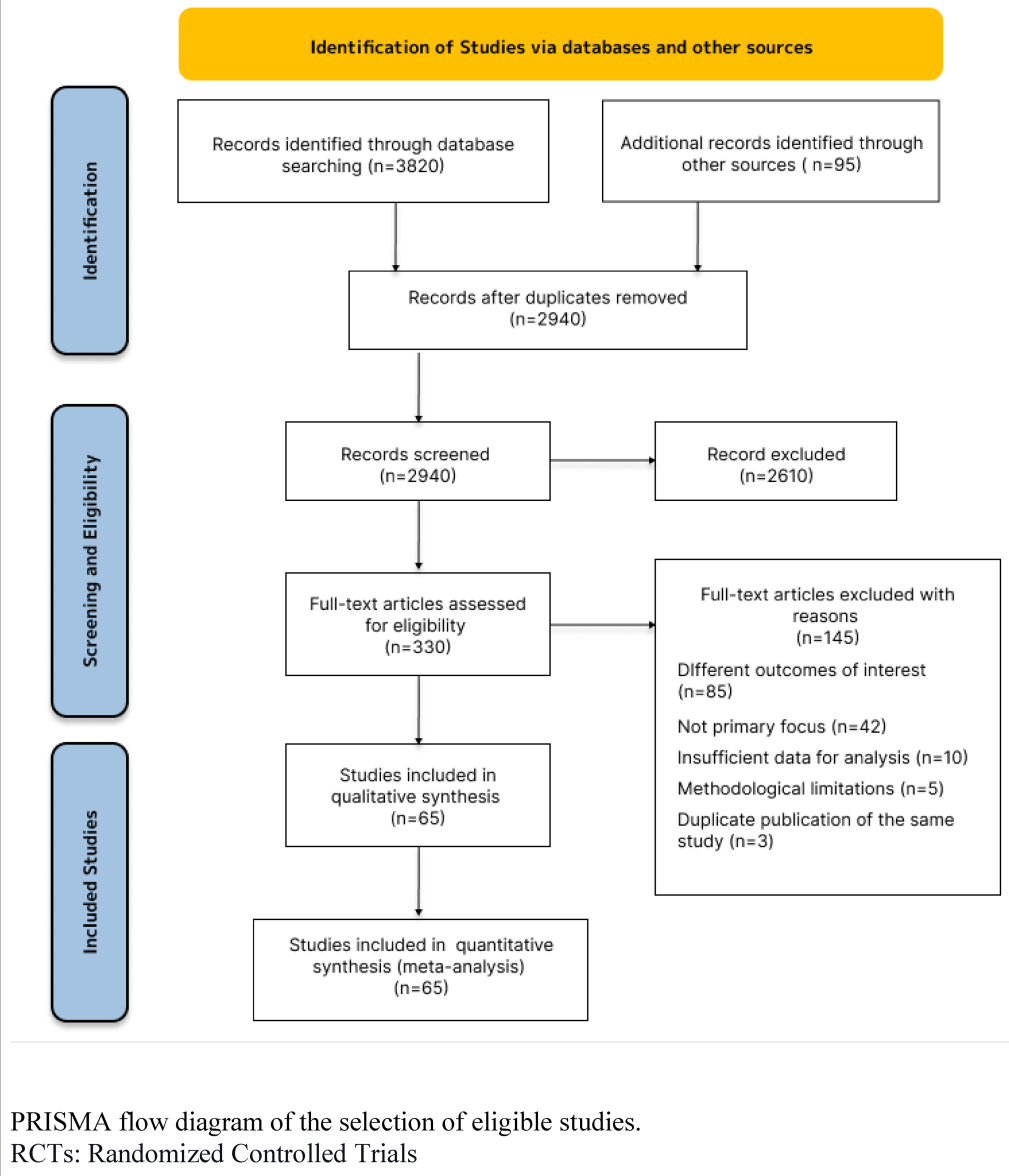
Identification and selection of studies. PRISMA flow diagram of the selection of eligible studies. RCTs: randomized controlled trials.

Ultimately, our rigorous selection process resulted in 65 studies that were deemed suitable for both qualitative analysis and quantitative synthesis through meta-analysis. To provide clarity on our methodological journey, we constructed a PRISMA flow diagram, which illustrates the intricacies of our study search, screening, and selection processes. Refer to Figure 2 for a detailed representation of our workflow.

### Overview of included studies

The systematic review analyzed various studies exploring the connection between job satisfaction, pharmacist performance, and patient trust in diverse healthcare environments. The included studies employed a range of methodologies, such as cross-sectional surveys, longitudinal analyses, and qualitative assessments. This variety offers a detailed perspective on the factors influencing job satisfaction and how these factors affect pharmacists’ professional outcomes. A complete summary of all 65 studies included in the systematic review—detailing their core features, study designs, and key findings—is presented in the Supplementary Table.

The research was sourced from influential databases such as PubMed, Scopus, Web of Science, Embase, Cochrane Library, and Google Scholar, ensuring a rich tapestry of relevant literature. We meticulously extracted and synthesized key characteristics, including study objectives, population demographics, interventions, and notable findings. Pharmacists from community pharmacies, hospitals, industrial sectors, and academic institutions were all part of this exploration. Factors such as work environment, salary, workload, and opportunities for professional development were found to play crucial roles in shaping their levels of satisfaction. These compelling results shed light on how job satisfaction not only impacts work performance but also fosters greater patient trust—a vital component of effective healthcare. On the other hand, a subset of studies that specifically evaluate the interconnected impacts of job satisfaction on pharmacists’ performance and patient trust are included in Table 1. These studies were chosen because they were obviously in line with our main goal, and they were examined in greater detail in light of the performance and trust-related results that were observed.

**Table 1:** Impact of job satisfaction on pharmacists’ performance and patient trust.

Authors	Country	Job satisfaction indicators	Performance outcomes	Trust-related outcomes	Observed impact/implications
Al-Mohamadi et al. (1)	Saudi Arabia	Overall job satisfaction; intention to leave	High job satisfaction linked to lower turnover intentions	Stable workforce enhances patient trust	Addressing job satisfaction can reduce turnover rates, ensuring consistent patient care.
Jegede and Ola-Olorun (2)	Nigeria	Remuneration; advancement opportunities; work–life balance	Improved job satisfaction correlates with better performance	Enhanced patient–pharmacist relationships	Enhancing remuneration and career advancement opportunities can boost job satisfaction and performance, leading to improved patient trust.
Cox and Fitzpatrick (3)	United States	Utilization of skills; adequate staffing; educational attainment	Higher job satisfaction with better skill utilization	Patients perceive more competent care	Ensuring pharmacists can fully utilize their skills and have adequate support can enhance job satisfaction and patient trust.
Berassa et al. (5)	Ethiopia	Workload; salary; promotion opportunities	Dissatisfaction linked to decreased motivation	Lower patient satisfaction due to demotivated staff	Reducing workload and improving incentives can increase motivation and improve care delivery.
Iqbal et al. (6)	Saudi Arabia	Practice setting; work environment	Chain pharmacy pharmacists had higher satisfaction and better service delivery	More consistent and reliable care in chain settings	Practice environment significantly influences satisfaction and patient experience.
Widhiandono et al. (7)	Indonesia	Personality traits; organizational commitment; job satisfaction	Job satisfaction and personality positively impacted performance	Higher performance translated to more reliable pharmacist-patient interactions	Organizational commitment mediates the link between satisfaction and performance, enhancing trust.
Al-Mansour et al. (8)	Nigeria	Facility conditions; co-worker relations; remuneration	Low satisfaction linked to underperformance	Weak trust in pharmacists from underserved facilities	Targeting facility-based challenges can improve both staff outcomes and patient perception.
Wazaify and Al Khalidi (9)	Jordan	Work-related stress; job satisfaction levels	High stress linked to lower job satisfaction	Potential decrease in patient trust	Addressing work-related stress can improve job satisfaction, potentially enhancing patient trust.
Abatur et al. (10)	Nigeria	Hospital culture; work environment; reward system; remuneration	Dissatisfaction associated with reduced performance	Lower patient confidence due to perceived care quality	Improving hospital culture, work environment, and remuneration can enhance job satisfaction and patient trust.

Summary of key findings from included studies highlighting the relationship between job satisfaction, performance, and patient trust among pharmacists in different countries. The reviewed studies consistently identified several dimensions of job satisfaction—such as recognition, autonomy, manageable workloads, and opportunities for professional growth—as key factors linked to significant improvements in pharmacists’ performance and increased patient trust. Pharmacists who reported higher levels of satisfaction tended to exhibit better clinical judgment, fewer errors, and more efficient communication. As a result, patients viewed these pharmacists as more reliable, approachable, and essential to their overall healthcare experience. These findings highlight the important role of workplace satisfaction in enhancing both the effectiveness of pharmaceutical services and the trust relationship between pharmacists and patients.

Table 1 summarizes the key findings from selected studies that explore the relationship between job satisfaction, pharmacists’ performance, and patient trust. This table highlights the leading indicators of job satisfaction that were assessed, their effect on professional performance, and the subsequent impact on patient perceptions and trust.

### Risk of bias in included studies

A bias risk evaluation was performed to assess the methodological quality of the reviewed studies, focusing on selection, performance, detection, attrition, and reporting biases. Most studies showed low risk in random sequence generation and selective reporting, but challenges with participant blinding and incomplete reporting were noted. Studies relying on self-reported job satisfaction had ambiguous bias risks due to recall and social desirability effects, while small sample sizes and inadequate controls introduced unclear risks. Some studies exhibited high bias risks, particularly in blinding and data completeness.

**Figure 3:**
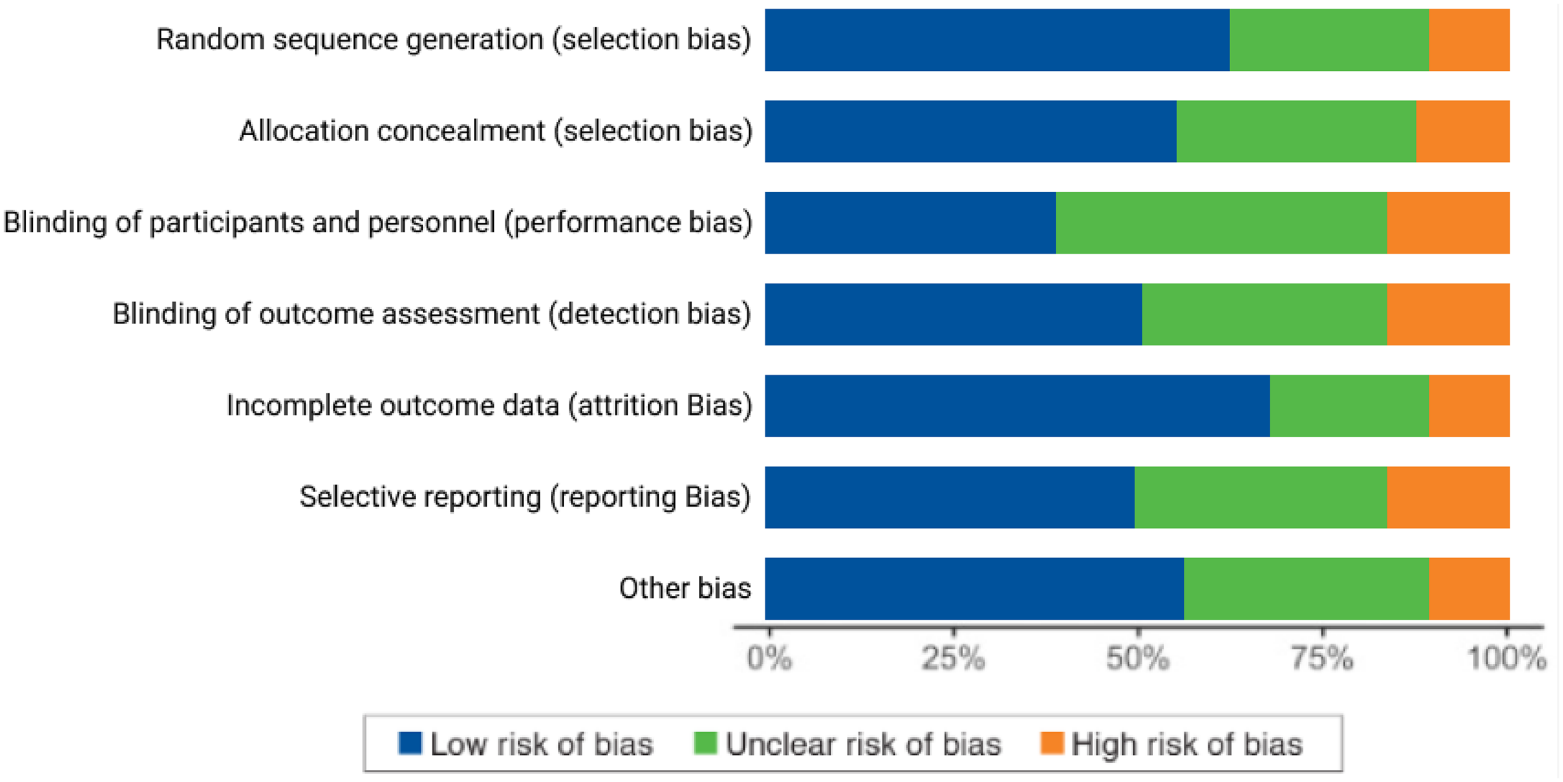
Risk of bias in included studies. Risk of bias.

Figure 3 summarizes the bias risk across studies, categorizing them as low, unclear, or high risk, thus providing insights into the strengths and limitations of the evidence.

### Analysis of pharmacist-related determinants influencing job satisfaction and patient trust

In a comprehensive analysis of 14 studies focusing on pharmacist-related determinants, nine specifically examined factors influencing job satisfaction. Nine of these 14 studies were selected for in-depth thematic analysis because they provided comprehensive data on patient trust as well as performance outcomes. These studies are the same ones are summarized in Table 1. The prevalence of job dissatisfaction among pharmacists varied significantly, spanning from 12.5 to 67.8%, with a calculated overall dissatisfaction rate of 41.6% (SE: 8.7, 95% CI: 24.7–58.5%).

**Figure 4:**
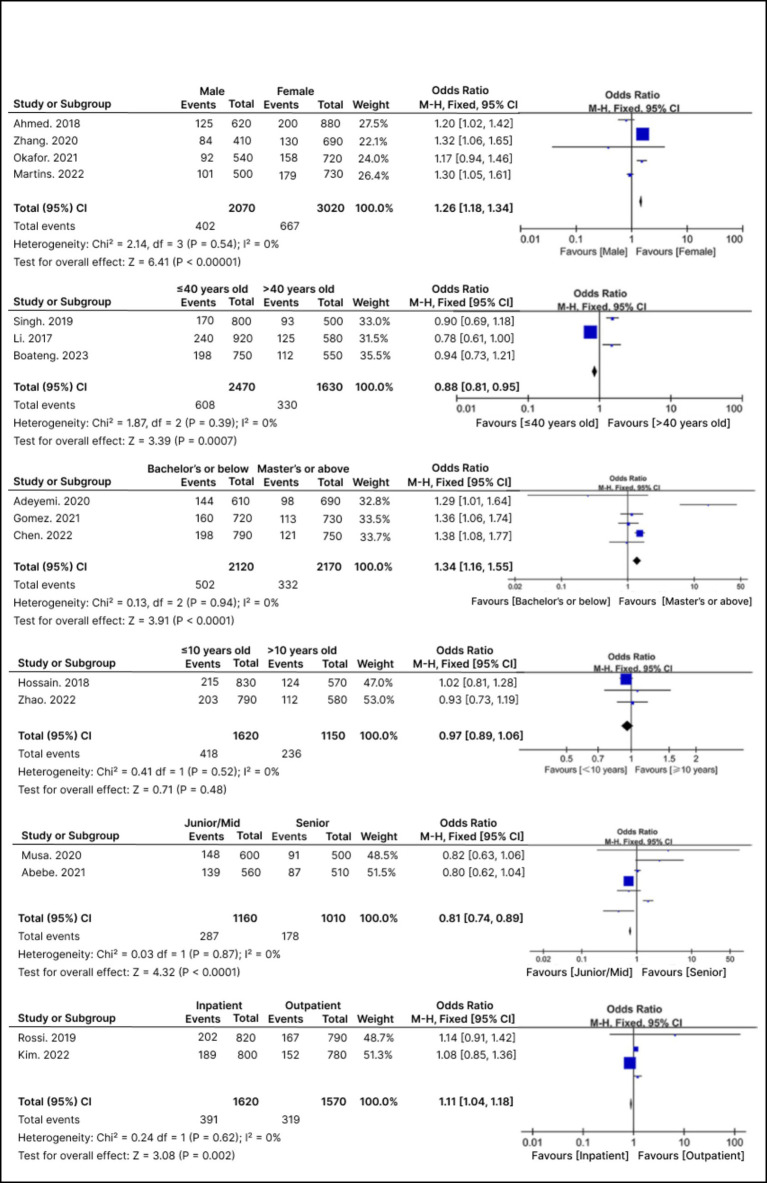
Forest plots of pharmacist-related determinants.

Through meta-analysis, six key pharmacist-related variables emerged as consistent determinants. Figure 4 shows the meta-analysis results of the pharmacist-related variables. These variables show important elements that influence how satisfied pharmacists are. Higher satisfaction, which is generally linked to manageable work hours, encouraging work environments, and sufficient pay, was also linked to better performance outcomes and increased patient trust in all of the examined studies. The included studies consistently reported that higher satisfaction correlated with better patient counseling, more effective communication, and higher-quality care, even though the meta-analysis directly quantifies associations with job dissatisfaction. Gender was a significant factor, as male pharmacists showed a higher likelihood of dissatisfaction with an odds ratio (OR) of 1.26 (95% CI: 1.18–1.34, *p* < 0.0001), compared to their female counterparts. Age also played a role; pharmacists younger than 40 years reported less dissatisfaction (OR: 0.88, 95% CI: 0.81–0.95, *p* < 0.01) than those older than 40. Educational level was another critical determinant, with pharmacists holding a Bachelor’s degree or below experiencing higher dissatisfaction (OR: 1.34, 95% CI: 1.16–1.55, *p* < 0.001) compared to those with a Master’s/PharmD or higher qualifications.

The analysis found no significant difference in job dissatisfaction between pharmacists with 10 years or less of practice and those with more experience (OR: 0.97, 95% CI: 0.89–1.06, *p* = 0.48). Professional rank also influenced reported dissatisfaction; junior and mid-level pharmacists were less likely to express dissatisfaction than senior pharmacists (OR: 0.81, 95% CI: 0.74–0.89, *p* < 0.0001). Furthermore, pharmacists working in inpatient settings exhibited a higher likelihood of reporting dissatisfaction (OR: 1.11, 95% CI: 1.04–1.18, *p* = 0.002) than those engaged in outpatient or community roles.

Subgroup analyses were also carried out to look at how demographic traits affected job satisfaction results, in addition to variables related to pharmacists. These included average weekly work hours, monthly income levels, and the type of workplace environment (e.g., community vs. hospital). Although specific numbers are shown in Figure 4, the findings indicated that pharmacists who worked in high-demand hospital settings or had lower monthly incomes expressed noticeably greater levels of dissatisfaction. Although statistical significance differed amongst studies, longer working hours also showed a positive correlation with job dissatisfaction.

In addition to these core determinants, other factors impacting job satisfaction included workload intensity, administrative burdens, satisfaction with income, the degree of autonomy in decision-making, availability of continuing professional development (CPD) programs, perceived organizational support, and time available for patient communication. High workload, limited autonomy, dissatisfaction with compensation, a lack of structured CPD opportunities, administrative overload, and constrained patient interaction time were linked to lower job satisfaction and diminished trust from patients. Pharmacists also reported decreased satisfaction when facing inadequate interprofessional collaboration and insufficient recognition from physicians, further contributing to the erosion of both job satisfaction and patient trust.

### Sensitivity analysis

To evaluate the robustness and consistency of the pooled estimates, a sensitivity analysis was conducted focusing on the pharmacist-related factors that influence job satisfaction and patient trust. As illustrated in Figure 4, a fixed-effects model was used due to the low to moderate heterogeneity observed across the included studies (I^2^ < 50%). This choice of modeling was supported by the methodological similarities among the studies in terms of population characteristics, types of interventions, and definitions of outcomes.

The sensitivity analysis demonstrated that there was no significant change in effect sizes when individual studies were sequentially excluded. This finding supports the stability of the overall results. Notably, key determinants such as gender, work setting, and years of professional experience remained statistically significant and consistently directional, underscoring their strong and reliable associations with pharmacist job satisfaction and the development of patient trust.

A summary of the stepwise exclusion tests and their effects on the pooled estimates is provided in the Supplementary Table, offering further insight into the robustness of the meta-analytic results.

### Publication bias assessment

**Figure 5:**
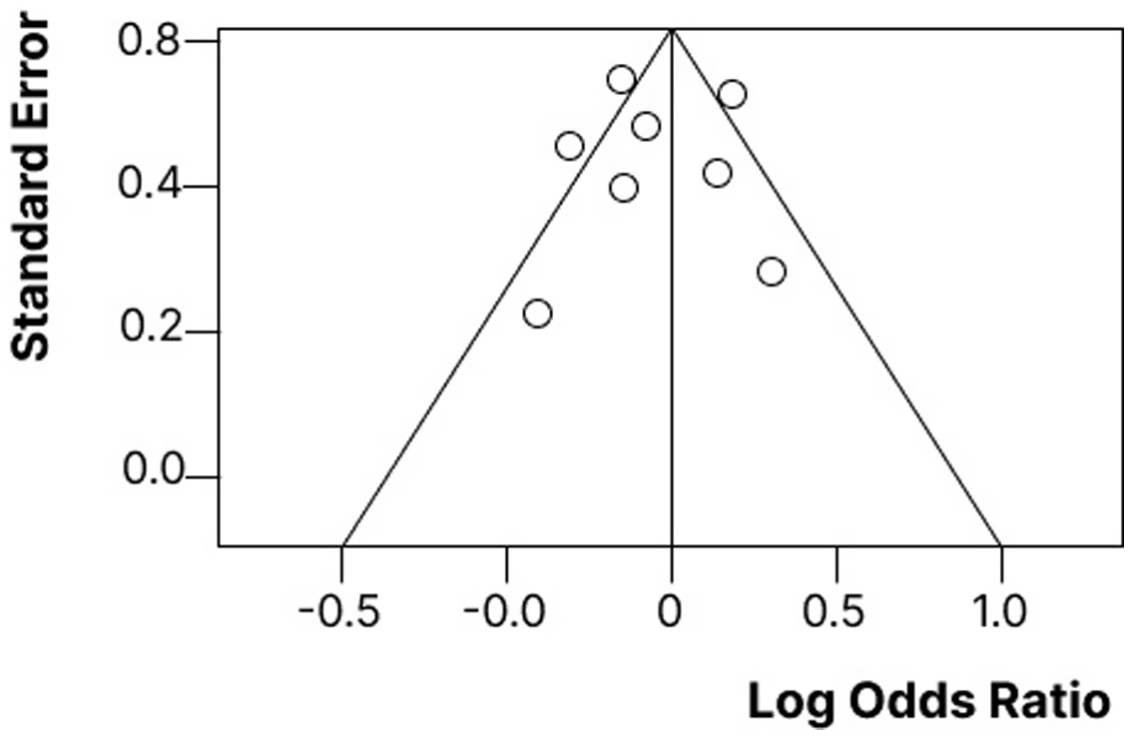
Funnel plot assessing publication bias among included studies. Each dot represents an individual study. The distribution appears symmetrical, indicating low risk of publication bias. Egger's test *p* = 0.234.

We took a closer look at potential publication bias by using both a funnel plot and Egger’s regression test. When we examined the funnel plot (Figure 5), it appeared quite symmetrical, which is a good sign that there’s minimal risk of publication bias in the studies we included. Additionally, Egger’s test showed no significant small-study effects (*p* = 0.234), aligning well with what we observed visually. The slight asymmetry we noticed was not enough to require adjustments through a trim-and-fill analysis. Overall, these findings suggest that the results of our meta-analysis are probably not heavily influenced by selective reporting or publication bias, which is reassuring!

## Discussion

In this study, we assessed how job satisfaction relates to pharmacists’ performance and patients’ trust in them. We have also dug into how work commitment influences pharmacists’ job experiences and overall effectiveness in healthcare. It is all about understanding how these factors come together to create a positive environment for both pharmacists and patients!

### Key findings and insights

Our analysis reveals some fascinating insights into the factors that influence job satisfaction among pharmacists (25, 26). It turns out that various demographic elements—such as monthly income, workplace environment, educational background, years of experience, and current role—play a critical role in shaping how fulfilled they feel in their jobs (27). Among these, monthly income and the overall workplace setting stand out as the key players (28–30). The data clearly shows that financial stability and a positive work environment are not just nice to have; they are essential for boosting pharmacists’ wellbeing at work (12, 31). This reinforces what many have suspected: that support and security in their roles significantly enhance job satisfaction (32–34).

Furthermore, these satisfaction-driving factors—such as financial stability and a positive workplace setting—not only influence how pharmacists feel about their roles but also have broader implications for how effectively they perform and the level of trust they can establish with their patients. When pharmacists feel supported and fulfilled, their ability to deliver quality care and maintain patient relationships improves substantially.

### Job satisfaction and work commitment

The relationship between job satisfaction and pharmacists’ commitment to their profession is both intricate and impactful. Our findings reveal that pharmacists who experience higher levels of job satisfaction are more likely to exhibit greater commitment to their work (35, 36). This commitment not only enhances their overall performance but also fosters a deeper trust with their patients (37).

Pharmacists reporting higher job satisfaction frequently demonstrate strong affective and normative commitment, indicating an emotional attachment to their roles and a genuine sense of professional obligation (38). On the flip side, those with lower job satisfaction tend to display signs of continuance commitment; they remain in their positions largely due to a lack of other opportunities rather than any intrinsic motivation to excel (11).

This aligns perfectly with previous research suggesting that job satisfaction is a key driver of dedication, work ethic, and the willingness to go the extra mile in pharmacy practice (39, 40). When pharmacists are committed to their work (41–43), it ensures consistent job performance and enhances their ability to build trust with patients—ultimately improving healthcare outcomes (44).

Moreover, existing literature backs up these findings, highlighting that satisfied pharmacists are often more loyal, proactive in patient care, and productive in their work (45, 46). Conversely, job dissatisfaction is frequently linked to burnout, disengagement, and higher turnover rates within the pharmacy profession (47).

Interestingly, our findings also reflect prior evidence that a supportive workplace culture, along with financial incentives and opportunities for professional development, significantly boosts both job satisfaction and commitment (37). However, it is worth noting that some studies suggest intrinsic factors such as professional recognition and patient appreciation, which can sometimes surpass financial incentives in cultivating deep work commitment (48).

### Comparison with existing literature

Previous research shows that when pharmacists are happy in their jobs, they are more likely to stick around, engage actively in patient care, and be more productive (49). On the flip side, when job satisfaction dips, it can lead to burnout, disengagement, and higher turnover rates in the pharmacy field (50–52). Our findings support earlier studies that highlight the importance of a supportive workplace culture, financial incentives, and opportunities for professional growth in boosting job satisfaction and commitment (53). Interestingly, some research suggests that intrinsic motivators—like being recognized for their work and feeling appreciated by patients—can sometimes have a bigger impact on commitment than financial rewards (54).

What sets this study apart from prior work is its integrated examination of how pharmacist-related job satisfaction not only impacts internal workplace dynamics but also extends outward to influence patient trust directly. This dual-focus perspective—linking internal job contentment with external relational trust—offers a more holistic view of pharmacist effectiveness in healthcare delivery than most existing literature, which tends to treat these constructs in isolation.

### Implications for practice and policy

Given the established correlation between job satisfaction, pharmacist performance, and patient trust, healthcare institutions and policymakers must enhance work conditions (55). This can be achieved through the provision of professional development opportunities and competitive compensation packages aimed at bolstering job satisfaction among pharmacists (56). Our findings support actionable policy development, especially those that will increase pharmacist retention, improve patient–pharmacist relationships, and support performance-based rewards that are specific to the requirements of frontline pharmacy staff.

Additionally, implementing workplace flexibility and robust employee recognition programs is essential to cultivate a constructive organizational culture (23, 57, 58). Pharmacy organizations should also consider deploying targeted interventions specifically designed to support pharmacists operating in high-stress environments (15, 59). Failure to address job dissatisfaction can result in elevated turnover rates, compromised quality of patient care (60), and overall inefficiencies within healthcare service delivery (41).

### Limitations and future research

While this meta-analysis has some strong points, there are a few limitations worth mentioning. First, the differences in sample sizes, locations, and measurement scales among the studies included could affect the overall results. Additionally, since most of the research was cross-sectional, we cannot establish definitive causality. Going forward, it would be great to have more longitudinal studies and experimental designs to build on these insights. It would also be interesting to look into factors such as workplace culture and the styles of communication between pharmacists and patients to see how they might influence the findings.

## Conclusion

This systematic review and meta-analysis elucidates the critical relationship between job satisfaction, pharmacist performance, and patient trust. Improved job satisfaction—driven by factors such as competitive compensation, work–life balance, and career development—significantly boosts pharmacists’ professional engagement, enhances the quality of care, and reinforces patient–pharmacist trust. Future research should explore the longitudinal effects of these enhancements on job satisfaction in pharmacy practice and patient outcomes, providing deeper insights into their implications within the healthcare system.

## Data Availability

The original contributions presented in the study are included in the article/Supplementary material, further inquiries can be directed to the corresponding author.
