# Supplementary material for: Job satisfaction and its correlation with pharmacists’ performance and patient trust
Source: Front Med (Lausanne). 2025 Jul 25;12:1624990. doi: 10.3389/fmed.2025.1624990 (PMC12331650; doi:10.3389/fmed.2025.1624990)
Supplement: Supplementary file 1 [file Table_1.docx]

## **Additional Information**

| Supplementary material [DOC-20250407-WA0012..docx](https://eu.docs.wps.com/module/common/preview/?sid=sIBvE7rroAa-Dm8MG) |
| --- |

| Supplementary material [DOC-20250407-WA0012..docx](https://eu.docs.wps.com/module/common/preview/?sid=sIBvE7rroAa-Dm8MG) |
| --- |

| Supplementary appendices [DOC-20250407-WA0012..docx](https://eu.docs.wps.com/l/sIMXW4MLiAczIz78G?v=v2) |
| --- |

| Supplementary appendices [DOC-20250407-WA0012..docx](https://eu.docs.wps.com/l/sIJ3W4MLiAeu31r8G?v=v2) |
| --- |
